# *Heading Date 3a* Stimulates Tiller Bud Outgrowth in *Oryza sativa* L. through Strigolactone Signaling Pathway

**DOI:** 10.3390/ijms251910778

**Published:** 2024-10-07

**Authors:** Qiqi Zheng, Zejiao Zhou, Xinran Li, Yingshan Lan, Ruihua Huang, Shengchun Zhang, Hongqing Li

**Affiliations:** 1 Guangdong Provincial Key Lab of Biotechnology for Plant Development, School of Life Sciences, South China Normal University, Guangzhou 510631, China; 19025279867@163.com (Q.Z.); 2016010131@m.scnu.edu.cn (Z.Z.); 202302292@m.scnu.edu.cn (Y.L.); huangruihua@m.scnu.edu.cn (R.H.); 2 State Key Laboratory for Conservation and Utilization of Subtropical Agro-Bioresources, South China Agricultural University, Guangzhou 510642, China; xrli@stu.scau.edu.cn; 3 Guangdong Laboratory for Lingnan Modern Agriculture, College of Life Sciences, South China Agricultural University, Guangzhou 510642, China

**Keywords:** *Heading date 3a*, *Oryza sativa* L., strigolactone signaling, tillering, proximity-dependent biotin identification

## Abstract

*Heading date 3a (Hd3a*, a *FLOWERING LOCUS T* (*FT*) ortholog from rice) is well known for its important role in rice (*Oryza sativa* L.), controlling floral transition under short-day (SD) conditions. Although the effect of *Hd3a* on promoting branching has been found, the underlying mechanism remains largely unknown. In this report, we overexpressed an *Hd3a* and *BirAG* (encoding a biotin ligase) fusion gene in rice, and found that early flowering and tiller bud outgrowth was promoted in *BHd3aOE* transgenic plants. On the contrary, knockout of *Hd3a* delayed flowering and tiller bud outgrowth. By using the BioID method, we identified multiple Hd3a proximal proteins. Among them, D14, D53, TPR1, TPR2, and TPRs are central components of the strigolactone signaling pathway, which has an inhibitory effect on rice tillering. The interaction between Hd3a, on the one hand, and D14 and D53 was further confirmed by the bimolecular fluorescence complementation (BiFC), yeast two-hybrid (Y2H), and co-immunoprecipitation (Co-IP) methods. We also found that Hd3a prevented the degradation of D53 induced by rac-GR24 (a strigolactone analog) in rice protoplasts. RT-qPCR assay showed that the expression levels of genes involved in strigolactone biosynthesis and signal transduction were altered significantly between WT and *Hd3a* overexpression (*Hd3aOE*) or mutant (*hd3a*) plants. *OsFC1*, a downstream target of the strigolactone signaling transduction pathway in controlling rice tillering, was downregulated significantly in *Hd3aOE* plants, whereas it was upregulated in *hd3a* lines. Collectively, these results indicate that *Hd3a* promotes tiller bud outgrowth in rice by attenuating the negative effect of strigolactone signaling on tillering and highlight a novel molecular network regulating rice tiller outgrowth by *Hd3a*.

## 1. Introduction

Rice (*Oryza sativa* L.) is the staple food for more than half of the world’s population. Rice grain yield is largely determined by four main components: the number of panicles per plant, number of grains per panicle, spikelet fertility, and grain weight [1,2]. Tillering in rice is an important trait of rice plant architecture which determines the number of panicles per plant. Flowering has a pivotal role in the developmental process determining effective panicle number, panicle size, and rice total yield. *FLOWERING LOCUS T (FT)*, which encodes the Phosphatidyl Ethanolamine Binding Protein (PEBP) as a major component of florigen, plays an important role in promoting floral transition through the photoperiod pathway. Rice is a facultative short-day (SD) plant, and two florigen genes, *Heading date 3a (Hd3a)* and *RICE FLOWERING LOCUS T 1 (RFT1)*, exert a major effect on rice heading date. These genes promote floral transition under SD and long-day (LD) conditions, respectively [3]. Like FT in Arabidopsis, Hd3a is produced in rice leaves and transported to SAM, where it forms the florigen activation complex (FAC) with 14-3-3 proteins of the GF14 family and bZIP transcription factor OsFD1. The complex is capable of inducing *OsMADS14* and *OsMADS15* expression and exerting an impact on the transition of SAM from the vegetative to reproductive stage [4]. Tsuji et al. [5] suggested that interaction between Hd3a and 14-3-3 proteins, but not OsFD1, is necessary to activate rice branching. However, the molecular mechanism of Hd3a in regulating rice branching remains to be elucidated.

Recent evidence suggested that the formation and maintenance of axillary buds in rice are regulated by multiple tillering-related genes such as *OsMOC1*, *OsMOC3*, *OsFON1*, *OsTB, OsTAD1*, etc. [6,7,8]. Strigolactones (SLs) are carotenoid-derived phytohormones and play important roles in regulating plant development processes, for example, in shaping plant architecture by inhibiting axillary bud outgrowth, affecting root development, responding to abiotic stress, etc. [9,10]. Notably, Visentin et al. [11] found that SLs promote flowering via induction of *SFT* (*SINGLE FLOWER TRUSS*, the homologue of *FT*), highlighting the role of SLs in plant reproduction development. Strigolactone signal transduction gene mutants exhibit dwarfism with increased tiller numbers, suggesting that SLs can negatively regulate tiller number in rice [12]. The central components of strigolactone signaling in rice are Dwarf 14 (D14), Dwarf 53 (D53), Dwarf (D3), and the TPR proteins referred to as TOPLESS (TPL) proteins. D14 is the receptor in strigolactone signal transduction [13,14]. Rice D14 mutant plants showed markedly increased tiller number and are insensitive to SLs [12]. D3 acts as a recognition subunit in the ubiquitin ligase complex (SKP1-CULLIN-F-BOX, SCF) and binds target proteins for 26S proteasomal degradation [15]. D53, as an inhibitor of strigolactone signal transduction, plays an important role in controlling axillary bud elongation. Additionally, D53 has been shown to act as a co-repressor in hormone signaling and plant development by interacting with TPL [16]. Degradation of D53 induced by SLs is dependent on the intact function of D3 and D14, and its protein stability affects the expression of *TEOSINTE BRANCHED1* (*OsTB1*, also named *OsFC1*), which is a downstream target of strigolactone signaling in controlling tiller growth [17].

In this study, we aimed at investigating the role of Hd3a in the molecular network regulating rice tiller outgrowth. Phenotypic comparison between *Hd3a* overexpression lines and knockout mutant plants revealed that altered *Hd3a* expression affects both flowering time and tiller bud outgrowth. We next screened Hd3a proximal proteins by applying the BioID system to rice protoplast [18], and we identified D14, D53, TPR1, TPR2, and TPR3 as potential candidates interacting with Hd3a. The interaction between Hd3a and D14 or D53 was further confirmed by molecular analysis, and that expression of *Hd3a* suppressed rac-GR24-induced D53 degradation in rice protoplasts. Finally, the transcription level of SLs’ synthesis and signal genes, including *OsD27*, *OsD3*, *OsD14*, *OsD53,* as well as downstream target *OsFC1,* were found to negatively correlate with *Hd3a* expression level in plants. These results indicated that *Hd3a* has a negative effect on tiller growth through inhibiting the strigolactone signaling pathway.

## 2. Results

### 2.1. Hd3a Promotes Heading Date and Tiller Bud Outgrowth

**Figure 1:**
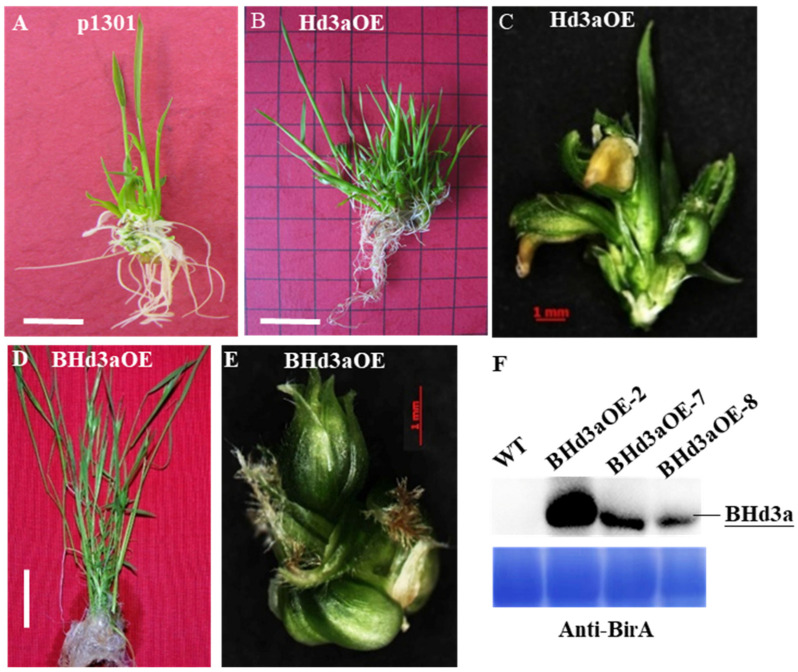
Overexpression of *Hd3a* (*Hd3aOE*) or *BirAG-Hd3a* (*BHd3aOE*) caused early flowering and bushy budding in in vitro culture. (**A**) Regenerated plantlets transformed with pCAMBIA1301 as a control. (**B**,**C**) Overexpressing *Hd3a* produced bushy floral shoots. (**B**) Abnormal floral organ. (**D**,**E**) Overexpression of *BHd3a* caused bushy floral shoots. (**D**) Abnormal floral organ. (**F**) Western-blot analysis of BirAG-Hd3a protein level in three lines. The scale bar in (**C**,**E**) is 1 mm, and the scale bar in (**A**,**B**,**D**) is 1 cm.

Overexpression of *Hd3a* (*Hd3aOE*) in rice causes extremely early flowering, which occurs as early as 30 days after embryogenic calli is transferred to regeneration medium. The calli is able to directly differentiate into floral structures, bypassing the route of normal bud development (Figure 1B) and forming incomplete florets with abnormal floral organs (Figure 1C). Compared with pCAMBIA 1301 transgenic (*p1301*) plants (Figure 1A), *Hd3aOE* transgenic buds grew in clumps (Figure 1B). Similarly, plants overexpressing *BirAG-Hd3a* fusion protein (*BHd3aOE*) also showed early flowering, abnormal floral organs, and bushy floral shoot phenotypes (Figure 1D, E). Floral structure formation and stem elongation developed simultaneously in bushy plantlets, with notable outgrowth of roots from elongation nodes during in vitro culture (Figure 1D). The results indicated that *Hd3a* promoted floral bud outgrowth in clumps, and *Hd3a* fused with *BirAG* did not influence *Hd3a*’s function in causing extremely early flowering and bushy bud formation. Unlike other *BHd3aOE* lines, *BHd3aOE-7 and BHd3aOE-8* set seeds normally, possibly attributing to their lower expression level of *Hd3a* (Figure 1F). Thus, *BHd3aOE-7 and BHd3aOE-8* were used to study the molecular mechanism of *Hd3a* in regulating tiller bud outgrowth in the subsequent experiments.

At the same time, we also generated *hd3a* null mutant lines via CRISPR/Cas9 technology (Figure S1A). On average, in paddy fields, WT plants (Zhonghua ‘11’) took 84 days until heading under long-day (LD) conditions and 69 days under short-day (SD) conditions. The flowering time for *BHd3aOE* plants was greatly reduced, to 30 days on average, under LD or SD conditions (Figure S1B,D,E). Compared with WT, *hd3a* lines delayed heading, on average, by 4.9 to 9.35 days under the LD condition and by 14.85 to 16.1 days under SD conditions (Figure S1D,E). The results indicated that knockout of *Hd3a* delayed flowering in contrast to extremely accelerated flowering in *BHd3a*.

**Figure 2:**
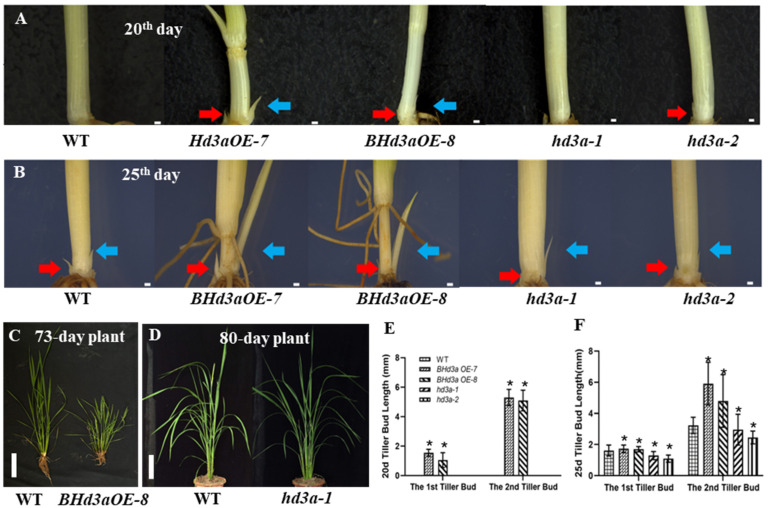
Rice tiller bud growth in *BHd3a* overexpression and *hd3a* mutation plants. (**A**) Close-up views of the basal region of tiller bud growth of WT, *BHd3aOE*, and *hd3a* plants at 20 days after sowing. (**B**) Close-up views of the basal region of tiller bud growth of WT, *BHd3aOE*, and *hd3a* plants at 25 days after sowing. (**C**) Gross morphology of WT and *BHd3aOE* at day73 after sowing. (**D**) Gross morphology of WT and hd3a at 80 days after sowing. (**E**) The first and second tiller bud length of WT, *BHd3aOE*, and *hd3a* plants at 20 days after sowing. (**F**) The first and second tiller bud length of WT, *BHd3aOE*, and *hd3a* at 25 days after sowing. Red and blue arrowheads indicate primary and secondary tiller bud, respectively. About 40 to 50 plants in each genotype were examined for tiller bud growth 20 or 25 days after sowing. Data are shown as means ± SD. “*” indicates significant differences, *p* < 0.05. The scale bar in (**A**,**B**) is 11 mm, and the scale bar in (**C**,**D**) is 10 cm.

Comparing tiller bud growth among different lines, we found that as early as the 20th day after sowing, lines overexpressing *BHd3a*, *BHd3aOE-7*, and *BHd3aOE-8* entered an elongation stage, with two tillers derived from the basal region of the main shoot and tillers from the node on elongated stem, whereas no visible tiller or internode was noted in WT plants or *hd3a* mutants, *hd3a-1* and *hd3a-2* (Figure 2A,E). On the 25th day after sowing, both WT and *hd3a* plants grew tillers hiding in leaves, but these were all shorter than those of the overexpression lines (Figure 2B,F). These results demonstrate that *Hd3a* promoted axillary bud outgrowth and accelerated tiller and internode elongation.

As shown in Figure 2C, 73-day *BHd3aOE* plants grew 11–14 tillers (panicles), which was more than the number grown by WT plants (5 to 8 tillers at the same time during the booting stage). No significant difference was found between WT plants (at the heading stage) and *hd3a* lines (not blossomed yet) regarding the number of tillers on day 80 (Figure 2D). Interestingly, WT rice plants generally showed weak apical dominance, with their main shoots becoming the highest shoot, and the later shoots growing shorter than the former shoot. On the contrary, in *BHd3aOE* plants, the later shoot or panicle of plants was observed to be taller or longer than the former. Overexpression of *Hd3a* contributes to attenuation of the apical dominance effect in rice. As shown in Figure S1C, in a 112-day *BHd3aOE* plant, the last shoot reached nearly the height of the WT plant’s main shoot. Inversely, *hd3a* plants were taller than WT plants, on average: plant height increased by 7.69 to 11.76 cm (Figure S1F). However, although *BHd3aOE* plants had more panicles compared with WT plants, *BHd3a* plants significantly reduced grain weight per plant due to their small panicles, caused by extremely early flowering (Figure S1G). In contrast, *hd3a* plants demonstrated increased grain weight per plant due to their large panicles, caused by late flowering. Overall, WT, *BHd3aOE*, and *hd3a* exhibited absolutely different tillering architectures. The results indicated that *Hd3a* hastened axillary bud outgrowth and its maturation, leading to a continuous flowering feature but with reduced grain yield. On the contrary, knockout of *Hd3a* delayed axillary bud outgrowth but enhanced plant height and grain yield.

### 2.2. Identification of Hd3a Proximal Protein with BioID

**Figure 3:**
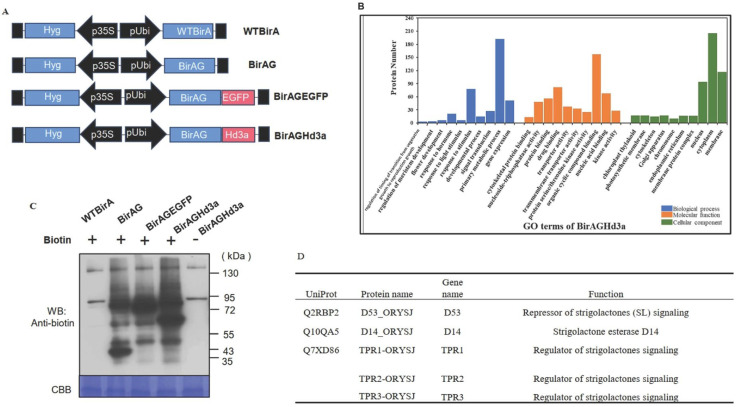
Screening of Hd3a proximal proteins with the BioID system in rice protoplasts. (**A**) Schematic map of control and BirAGHd3a constructs. (**B**) GO terms of Hd3a proximal proteins. (**C**) Western blot shows that the BirAGHd3a works efficiently in rice protoplasts. (**D**) The central components for strigolactone signaling identified in Hd3a proximal proteins.

To dissect the molecular network of *Hd3a*’s regulation on tiller bud growth, proximal proteins with Hd3a were identified using the BioID system in rice protoplast. For this purpose, expression vectors of WTBirA, BirAG, BirAGEGFP, and BirAGHd3a were constructed based on the binary vector pCAMBIA1390 [18] (Figure 3A). We then investigated whether BirAGHd3a can efficiently biotinylate proteins in rice cells. Rice protoplasts were transformed with the four vectors, respectively. After 24 h incubation of transformed protoplasts with or without 50 mM biotin, proteins were extracted and examined by Western blot assay (Figure 3C). In the presence of biotin, biotinylation of multiple proteins in rice protoplasts was detected in protoplasts transformed with BirAG, BirAGEGFP, and BirAGHd3a. On the contrary, no additional biotinylated bands were detected in protoplasts transformed with WTBirA or BirAGHd3a without the addition of biotin. These findings are similar to our previous results whereby BirAG alone, or fused with target proteins, was observed to function efficiently on its vicinal partners [18]. Compared with BirAG, the Western blot assay of BirAGHd3a showed a distinct band pattern of protein biotinylation, suggesting that BirAG-fused Hd3a has potential in detecting Hd3a-specific proximal proteins in rice.

We next performed MS/MS analysis of the biotinylated proteins isolated from protoplasts transformed with BirAHd3a, WTBirA, and BirAG and of the latter two as controls to remove background noise. In total, six MS/MS data lists were generated (two biological repeats were performed for each vector). After a MASCOT search of the rice reviewed proteins in the Uniprot database, 539, 477, and 667 proteins were found in WTBirA, BirAG, and BirAGHd3a, respectively. Comparison of these proteins revealed that 213 proteins presented exclusively in BirAGHd3a datasets, which constituted the largest component of the Hd3a proximal proteins (Supplemental dataset S1).

We classified these Hd3a proximal proteins by GO analysis. The protein GOSlim was assigned by batch download from the database RAP-DB (Rice Annotation Project) and categorized into BP biological process (BP), molecular function (MF), and cellular component (CC) (Figure 3B). Proteins in the molecular function category are clustered by their regulation of the time of transition from vegetative growth to reproductive growth and by their regulation of meristem development, flower development, and response to hormone. Proteins in the cellular compartment category are observed to be distributed in cytoplasm, membrane, and nucleus. Enriched proteins in the biological processes category are widely involved in protein binding, nucleic acid binding and organic cyclic binding (Figure 3B). Notably, a number of 14-3-3 proteins were enriched in interacting protein. These proteins were previously reported to interact with Hd3a. The results indicated that these proteins are widely involved in regulating multiple developmental processes, flower development, meristem development, the timing of transition from vegetative to reproduction growth, etc., and again addressed the fact that Hd3a plays an important role in controlling floral transition.

### 2.3. Hd3a Promotes Tiller Bud Outgrowth by Suppressing Strigolactone Pathway

**Figure 4:**
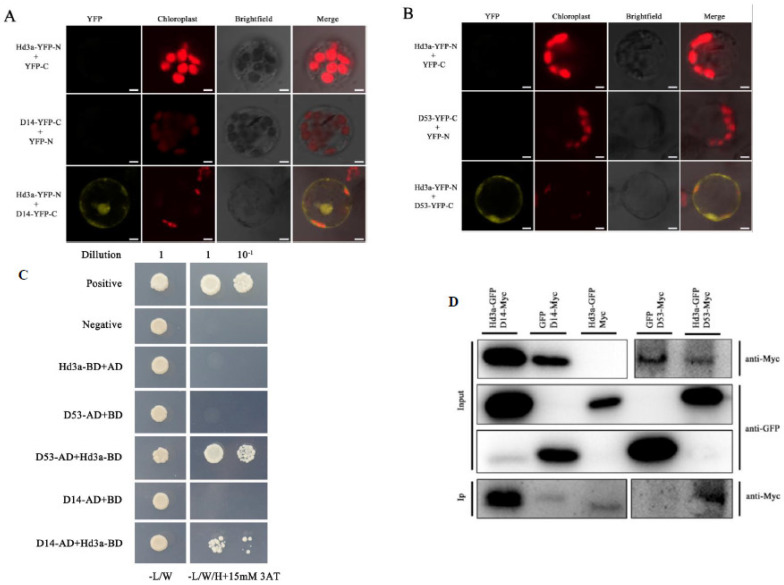
Molecular analysis of Hd3a interaction with D14 and D53. (**A**) BiFC verified the interaction between Hd3a protein and D14 protein in rice protoplasm. Hd3a was fused with nYFP; D14 was fused with cYFP. (**B**) BiFC verified the interaction between Hd3a protein and D53 protein in rice protoplasm. Hd3a and nYFP were fused; D53 and cYFP were fused. The scale is 5 mm. YFP: yellow fluorescence signal. (**C**) Yeast two-hybrid verification of Hd3a protein interaction with D14 or D53. The co-transformed yeast strains on SD-2 (SD/-Trp/-Leu) medium were inoculated on SD-3 (SD/-Trp/-Leu/-His) medium, and the experimental results were observed and photographed. Dilution: yeast solution dilution ratio; -L/W: SD-2 (SD/-Trp/-Leu) medium; -L/W/H: SD-3 (SD/-Trp/-Leu/-His) medium; 3AT: 3-amino-1,2,4-triazole. (**D**) Co-immunoprecipitation verification of Hd3a protein interaction with D14 or D53. The experimental group (Hd3a-GFP + D14-Myc and Hd3a-GFP + D53-cYFP) and the control group (GFP + D14-Myc, Hd3a-GFP + Myc, and GFP + D53-Myc) were co-transformed into rice wild-type protoplasts, respectively. After 12 h culture under dark conditions, the proteins were extracted, and Western blotting was performed using anti-GFP antibody and anti-MyC antibody.

Strigolactone is one of the most important hormones that can inhibit axillary bud outgrowth in rice, for instance, by regulating *OsFC1* expression [2,6,19]. In our study of Hd3a proximal proteins, five proteins were found to modulate the strigolactone signaling pathway, including D14_ORYSJ, D53_ORYSJ, TPR1-ORYSJ, TPR2-ORYSJ, and TPR3-ORYSJ (Figure 3D). We further carried out molecular analysis to confirm the interaction between Hd3a, on the one hand, and D14 and D53. BiFC results suggested that the interaction between Hd3a, on the one hand, and D14 and D53 occurred mainly in the nucleus (Figure 4A,B). Yeast two-hybrid results showed that there is no interaction between Hd3a, on the one hand, and D53 and D14 (Figure 4C). Co-immunoprecipitation (coIP) results confirmed that Hd3a protein interacts with both D14 and D53 (Figure 4D).

**Figure 5:**
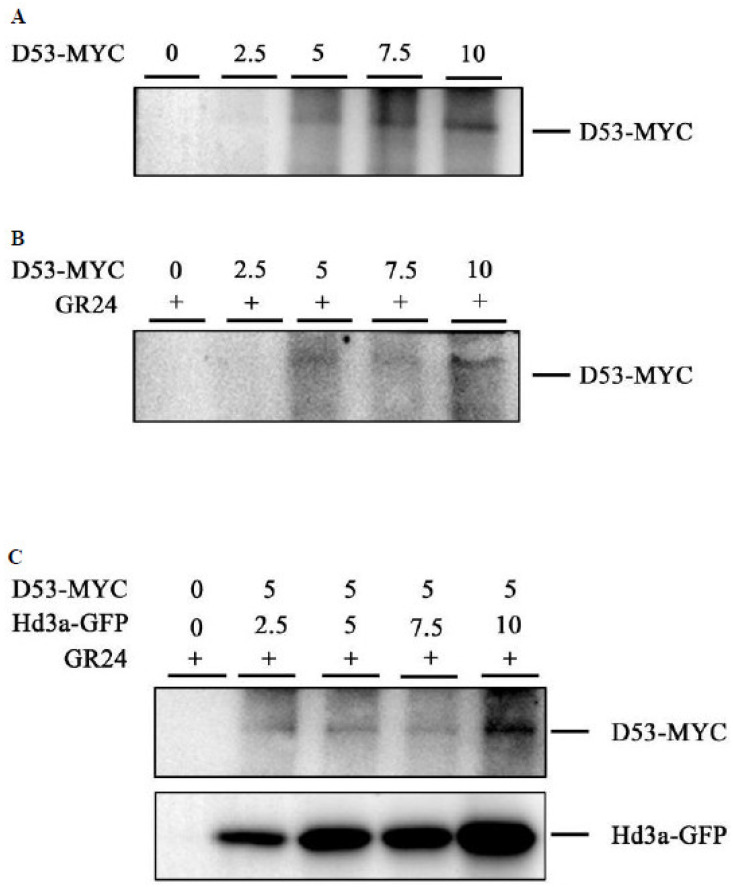
Hd3a inhibits rac-GR24-induced D53 protein degradation in rice protoplasts. (**A**) Expression of D53-Myc in rice protoplast; plasmid D53-MYC in the amount of 2.5 μg, 5 μg, 7.5 μg, and 10 μg were transformed into rice protoplasts, respectively, and incubated under dark conditions for 12 h. (**B**) rac-GR24-induced D53 degradation in rice protoplasts. After transformation, 1 μM rac-GR24 was added to the protoplast incubation buffer. (**C**) Hd3a inhibits rac-GR24-induced D53 degradation. Plasmids D53-MyC (5 μg) and Hd3a-GFP (2.5 μg, 5 μg, 7.5 μg, and 10 μg) were co-transformed into rice protoplasts, and rac-GR24 was added in the incubation solution. Protein levels of D53-MYC and Hd3a-GFP were detected using anti-MyC and anti-GFP antibodies.

In the presence of SLs, D14 is activated and interacts with D3 to form an SCF^D3^-type E3 ubiquitin ligase complex (SCFD3) which ubiquitinates D53 and facilitates its degradation by the 26S proteasome pathway and promotes downstream gene expression of *OsFC1*. Thus, the rate of D53 degradation is crucial in strigolactone signaling cascades. For this, we investigated whether the interaction of Hd3a with D14 and D53 interfered with D53 degradation in rice protoplasts. As shown in Figure 5A, transformation of increasing amounts (0, 2.5, 5, 7.5, and 10 µg) of the plasmids D53-MYC into rice protoplasts was able to produce protein gradients in each sample, as indicated by the increasing intensity of the D53-MYC bands. Supplementation of rac-GR24, a synthetic analog of strigolactones, into the transformed protoplasts caused the degradation of D53, which is a phenomenon in strigolactone signaling. However, during co-transformation of Hd3aGFP with D53-MYC, we observed that rac-GR24-induced D53 degradation was inhibited, and this effect was improved when the amount of Hd3aGFP in protoplasts was increased (Figure 5B). These results suggest that Hd3a plays a role in stabilizing D53.

**Figure 6:**
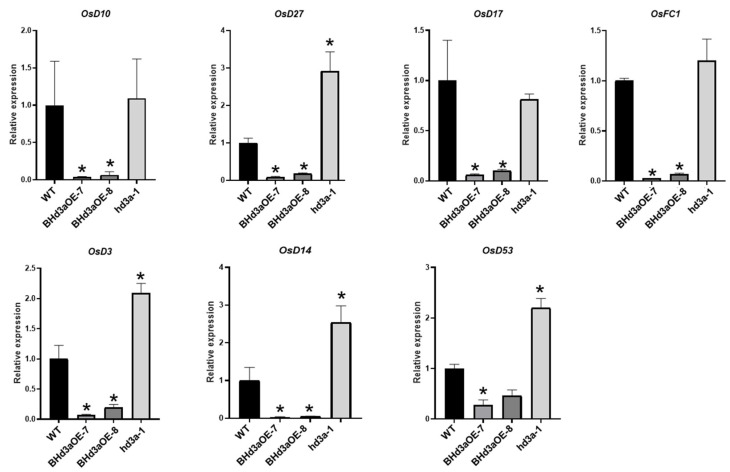
Transcription level of genes related to strigolactone synthesis and signaling. The relative expression levels of SL-synthesis-related genes *OsD10*, *OsD17*, and *OsD27* in WT plants, mutant *hd3a-1* plants, and plants overexpressing *Hd3aOE-7* and *Hd3aOE-8*. The relative expression levels of SL-signal-transduction-related genes *OsD3*, *OsD14*, and *OsD53* in WT plants, mutant *hd3a-1* plants, and plants overexpressing *Hd3aOE-7* and *Hd3aOE-8*. Each experiment was repeated three times. Data are shown as means ± SD. “*” indicates significant differences, *p* < 0.05.

To further explore Hd3a’s role in the strigolactone signaling pathway, we used RT-qPCR to investigate the gene expression level of three synthesized, SL-related genes (*OsD10*, *OsD17*, and *OsD27*), three genes related to strigolactone signaling transduction (*OsD3*, *OsD14*, and *OsD53*), and the strigolactone signaling pathway downstream gene (*OsFC1*). As shown in Figure 6, compared with WT plants, the expression level of all seven genes was significantly reduced in both *BHd3aOE* lines. On the contrary, the expression levels of *OsD27, OsD3*, *OsD14*, *OsD53*, and *OsFC1* were significantly increased in *hd3a-1*. There was no significant difference in both *OsD10* and *OsD17* expression levels between WT and *hd3a-1*. The results indicated that expression levels of Hd3a are negatively correlated with the expression of genes related to essential components of the strigolactone signaling pathway. Overall, our study demonstrated that *Hd3a* promotes tiller elongation and outgrowth by suppressing the strigolactone signaling pathway.

## 3. Discussion

In this study, we investigated the molecular mechanism of *Hd3a* in promoting rice tiller bud outgrowth. Using the BioID method, we identified Hd3a’s proximal proteins D14, D53, and TPRs, which are central components of the strigolactone signaling pathway. We further confirmed the interaction of Hd3a with D14 and D53 and found that Hd3a can suppress rac-GR24-induced D53 degradation in protoplasts. These results, together with the transcriptional assay of the strigolactone signaling-related gene expression, indicated that *Hd3a’s* role in promoting tiller outgrowth occurred via inhibition of the strigolactone signaling pathway.

### 3.1. Hd3a Promotes Flowering and Tiller Bud Outgrowth in Rice

Flowering time is an important agronomic trait for season adaption and grain yield, as early or late flowering can cause a serious reduction in production: early flowering produces small panicles due to insufficient vegetative growth, while late flowering results in poor flower development under cold temperature in autumn. As a key regulator of flowering time, *FT* always attracts a great deal of attention from researchers, with new elements in its role continuously being revealed. For instance, in addition to its function in promoting anthesis, *FT* regulates other essential developmental processes such as leaf morphology [20], tuber formation [21], bulb formation [22], the opening of stomatal guard cells [23], and tillering or branching patterns [5,24,25]. In this study, we also found that *Hd3a* stimulated calli to differentiate and develop short floral structures, bypassing the route of normal bud development in in vitro tissue culture (Figure 1). In a paddy trial, *BHd3aOE* plants exhibited extremely shortened heading dates (Figure 2). Compared with WT plants, overexpression of *Hd3a* led to continuous flowering in *BHd3aOE* plants, with its later shoot or panicle growing higher or longer than the prior one, contributing to bushy tillers/panicles in *BHd3aOE* plants. In addition to *Hd3a*’s role in stimulating flowering, it was found to impact normal reproductive development, for example, by producing incomplete or abnormal floral organs, busy floral bud outgrowth (Figure 1), and other developmental processes via delaying or inhibiting seed germination, thus improving rice drought and salt tolerance (our unpublished data). In this study, our results highlighted *Hd3a*’s role in promoting tiller bud outgrowth (Figure 2) and highlighted that varying *Hd3a* expression levels in different rice lines led to different plant architecture and yield. Furthermore, there is a trade-off between an early flowering time and panicle size, as well as between tillering and panicle size. Compared with WT plants, extremely early-flowering plants produced small panicles. This led to a loss of grain yield despite increased panicles in *BHd3aOE* plants (Figure 2). Although knocking out *hd3a* had no obvious effect on the number of panicles compared with WT plants in the same planting time, *hd3a* plants exhibited increased grain weight per plant and increased plant height. Therefore, as an important regulator of both tiller outgrowth and reproductive developmental processes, which directly determinate rice yield, *Hd3a* expression level should be finely tuned to avoid extremely early flowering and loss of rice yield [25].

Further understanding of *Hd3a*’s role in regulating plant development contributes to rice breeding. Taoka et al. [4] employed an FAC model to illustrate *Hd3a*’s regulation of flowering time in rice. Later, this model was adopted to clarify the roles of *Hd3a* in regulating inflorescence development in *Medicago truncatula* and pea [26,27,28], carpel development in rice [29], branching in rice [5], lateral root development in cotton [30], and bud seasonal growth and stress tolerance in trees [31,32]. Recently, Tsoy and Mushegian [33] proposed that *FT* and its related proteins are enzymes operating on small diffusible molecules, which may constitute an overlooked essential ingredient of the florigen signal. Thus, the molecular mechanism of *Hd3a* function remains insufficiently understood, and there is an urgent need to dissect the regulatory mechanism of *Hd3a* in regulating traits other than flowering time, for example, in regulating tiller bud outgrowth.

### 3.2. Hd3a Mediated Promotion of Tiller Bud Outgrowth through Strigolactone Pathway

Tillering is a determinant factor of plant architecture and yield in rice and is regulated by a complex network of genetic factors, plant hormones, and environmental factors [8]. Tillering in rice is shaped by axillary bud formation and axillary bud growth. In rice, *MONOCULM 1 (MOC1*), *MONOCULM 3* (*MOC3*), and *FLORAL ORGAN NUMBER 1* (*FON1*) have been found to control tiller bud formation and growth. It was demonstrated that MOC3 can bind to the promoter of *FON1* and activate its expression, thus stimulating tiller bud growth. MOC1 interacts with MOC3 and acts as a coactivator of MOC3 [7]. Plant hormones are also involved in the control of rice tiller bud growth. The strigolactone signaling protein D53 has a suppression effect on downstream *OsFC1* expression, promoting tiller bud growth. The BR signaling pathway plays an antagonist role against the strigolactone signaling pathway in regulating *OsFC1* expression and tiller bud growth. In the presence of BRs, the BR signaling components OsBZR1-RLA1-DLT and D53 suppress the expression of *OsFC1* [34]. Cytokinin is also involved in tiller bud control, as altered expression of cytokinin metabolism genes has an impact on tiller number [35,36]. Auxin, along with SLs and cytokinin, control tiller number and whether some auxin synthesis and transporter genes regulate tiller number [37]. Notably, Tsuji et al. [5] suggested that Hd3a interacts with 14-3-3, but not with OsFD1, to activate axillary meristem outgrowth in rice, and this process is independent of strigolactone, auxin, or cytokinin signaling.

In this study, we found that strigolactone-signaling-pathway-related proteins, D14, D53, TPR1, TPR2, and TPR3 were Hd3a proximal proteins to be isolated by BioID method. Interaction between Hd3a, on the one hand, and D14 and D53 were further confirmed by molecular approaches (Figure 4). It was shown that Hd3a might inhibit D53 degradation induced by SLs, resulting in attenuated strigolactone signal transduction and leading to downregulation of *OsFC1* and affecting tillering. The expression level of a number of SLs (biosynthesis, signaling genes, and downstream genes) in controlling tillering were found to be downregulated following *Hd3a* overexpression but upregulated in *hd3a* mutants. Thus, it is suggested that *Hd3a* impedes the effects of SLs in suppressing tiller bud outgrowth by repressing the D53 degradation and by downregulation of genes associated with the strigolactone signaling pathway.

Mutants associated with SLs’ biosynthesis and signaling pathway, such as D27, D17, D10, and D53, all exhibited highly branched and dwarf phenotypes in rice; however, information about the flowering times of these mutants is scarce. Recently, Visentin et al. [11] showed that SLs promoted flowering and affected flower development in tomato plants through the miR319-LA-SFT module, uncovering the relationship between SLs and *FT*. In our study, we shed new light on the interaction of *Hd3a* with SLs in regulating rice tiller bud outgrowth. Unlike the aforementioned floral transition in tomato plants, the interaction of *Hd3a* with SLs is not affected by the photoperiod. Rice is a short-day photoperiodic flowering plant, and *Hd3a* is the key component of controlling flowering time in response to photoperiod. Thus, it would be very interesting to further explore the roles of the strigolactone signaling pathway in regulating flowering time and tillering in rice. 

## 4. Materials and Methods

### 4.1. Vector Construction

Vectors for overexpression of Hd3a, Hd3aOE, and BHd3aOE were constructed based on pCAMBIA1390, in which the CaMV35S promoter was replaced by a maize ubiquitin promoter [38]. Vectors of WTBirA, BirAG, BirAGEGFP, and BirAGHd3a were constructed according to our previous paper [18]. Briefly, *BirA* and *BirAG* were amplified by polymerase chain reaction (PCR) from *Escherichia coli*. Then, *BirA*, *BirAG,* or *BirAG* fused with *EGFP* or *Hd3a* was incorporated into pCAMBIA1390, respectively.

The sgRNA-Cas9 plant expression vectors were kindly provided by Prof. Yaoguang Liu (South China Agriculture University). The vectors were constructed by inserting synthesized oligos into the BsaI site of the vector pYLCRISPR/Cas9(I), which contains a codon-optimized Cas9 driven by a maize ubiquitin promoter, a sgRNA scaffold directed by a rice U6a promoter, and a backbone of the binary vector pCAMBIA1300 (CAMBIA, Canberra, Australia) [39]. The oligos used in constructing the sgRNA vector for *Hd3a* is Hd3aC-Fw: GCCGACCTCGACCCTAGGCTGGT; Hd3aC-Re: AACACCAGCCTAGGGTCGAGGT.

### 4.2. Generation of Hd3aOE, BHd3aOE, and hd3a Plants

The constructs were introduced into *Agrobacterium tumefaciens* strain EHA105 by electroporation. *Agrobacterium*-mediated transformation of rice (*Oryza sativa* L. ssp. *japonica*. Zhonghua 11) was performed as described [40]. Briefly, embryogenic calli were infected with *Agrobacterium* and selected on hygromycin containing medium for callus proliferation for 30 days. Afterwards, the hygromycin-resistant calli were transferred to regeneration medium for shoot induction, and finally, shoots were transferred to root induction medium for rooting. Transgenic plants were confirmed by the detection of *Hd3a* expression level or by sequencing of targeting site afterPCR amplification.

### 4.3. Agronomic Trait Analyses

Wild-type (WT) and T3 generation homozygous lines of *BHd3aOE-7*, *BHd3aOE-8*, *hd3a-1*, and *hd3a-2* were planted in experimental fields, respectively, at South China Normal University in Guangzhou, P.R. China, with their phenotypes being investigated in 2022 and 2023, respectively. About 40 to 50 seeds of each genotype were sown on 16 March and 16 July each year. Since results generated from 2022 and 2023 are the same, only the representative data from 2022 were presented in the current study.

### 4.4. Protoplasts Isolation and Transfection

Rice protoplasts’ isolation and transfection were performed as described in our previous study [18]. Briefly, the stem and sheath tissues of rice seedlings were used to isolate protoplasts. An amount of 10 μL of plasmids at a concentration of 1 μg/μL was transfected to 100 μL (About 2 × 10^6^) of protoplasts. Then, the transfected protoplasts were collected and resuspended in 250 µL of WI. For biotinylation experiments, WI containing 50 µm of biotin was added to the protoplasts. For treatment with rac-GR24, WI containing 1 µm of rac-GR24 was added.

### 4.5. Western Blotting

About 1 × 10^6^ protoplasts were lysed in Laemmli SDS sample buffer. Proteins were separated by SDS-PAGE and transferred to PVDF (Roche, Basel, Switzerland) membrane [41]. Immunoblotting was performed [42] with different antibodies. For detection of biotinylated proteins, membranes were blocked in 3% casein in TBS with 0.05% Tween-20 and incubated in the same buffer with HRP-conjugated streptavidin (1:40,000; Invitrogen, Carlsbad, CA, USA).

### 4.6. Affinity Capture of Biotinylated Proteins

About 3 × 10^6^ protoplasts were transformed and incubated for 24 h in WI solution with 50 μM of biotin. After three washes with 0.6 M of mannitol solution, protoplasts were lysed according to [43]. The supernatant containing the extracted proteins was incubated with 600 μL of Dynabeads (MyOne Steptavadin C1; Invitrogen, Carlsbad, CA, USA) overnight at 4 °C. Stringent washing of beads was performed according to [43]. A total of 10% of the beads were reserved for Western blot analysis. Bound proteins were removed from the magnetic beads with 50 μL of Laemmli SDS-sample buffer saturated with biotin and boiled at 98 °C for 10 min.

### 4.7. Protein Identification Using Mass Spectrometry

Protein identification using mass spectrometry was conducted as described in [18]. After stringent washing, the beads were washed six times with 50 mM of ammonium bicarbonate (pH 8.3) and treated with 0.5 μg/μL of TPCK-trypsin (Promega, Madison, WI, USA) for 16 hrs at 37 °C. The supernatant containing the tryptic peptides was collected and lyophilized.

Nano LC MS/MS analysis was carried out using an Orbitrap Fusion Tribrid (Thermo-Fisher Scientific, San Jose, CA, USA) mass spectrometer according to the manufacturer’s protocol. Raw data files acquired from Fusion were converted into MGF files using Proteome Discoverer 1.4 (PD 1.4, Thermo). Subsequent database searches were carried out by Mascot Daemon (version 2.4.1, Matrix Science, Boston, MA, USA) for both protein identifications and TMT quantitation against the protein databases. Only peptides above “identity” were counted as identified, and it was required that each confident protein identification involved at least two unique peptide identifications indicated in Mascot. The proteins identified within the same family were grouped. For each construct, two biological repeats were performed.

### 4.8. BiFC Analysis of Putative Vicinal Proteins in Rice Protoplasts

D14 and D53 coding sequences were amplified with PCR and restriction-cloned (BamHI and/or EcoRI) into the p35S::cYFP vector, which was in fusion with the C-terminal fragment of YFP at the 3′ end. In the meantime, Hd3a coding sequence was PCR amplified and restriction-cloned into the p35S::nYFP vector in fusion with the N-terminal fragment of YFP at the 5′ end. All constructs were sequence-verified before transformation. The vectors were co-transformed into rice protoplasts and cultivated overnight before live imaging. Pictures were acquired using LSM 710 confocal microscope (Zeiss, Jena, Germany).

### 4.9. Yeast Two-Hybrid Analysis

Yeast two-hybrid analysis was performed using an AH109-GAL4 system (Clontech, Mountain View, CA, USA). PCR-amplified fragments of Hd3a were cloned into pGBKT7 (Y2H) to form the bait construct, and the fragments containing D14 and D53 were inserted into pGADT7 to form the prey constructs. The bait and prey constructs, together with thermally denatured salmon sperm carrier DNA, were transformed into yeast strain AH109 according to the manufacturer’s instructions (Clontech, Mountain View, CA, USA). Then, the transformants were grown on various media at 28 °C.

### 4.10. Quantitative RT-PCR

Tiller buds were sampled from 25-day old WT, *hd3a-1*, *Hd3aOE-7*, and *Hd3aOE-8* plants, respectively, frozen into liquid nitrogen, and stored at −80 °C before RNA extraction. RNA extraction from tiller buds was performed using Trizol reagent (Invitrogen, Carlsbad, CA, USA). RNA samples were treated with DNaseI and were quantified using Nanodrop Spectrophotometer (Nanodrop Technologies, Wilmington, NC, USA). First-strand cDNA synthesis and qRT-PCR (quantitative reverse transcriptase polymerase chain reaction) were performed according to the instructions of commercial kits (TransGen Biotech, Beijing, China). PCR amplification was performed using the following procedure: 30 s at 94 °C preincubation, 45 cycles of 5 s denaturation at 94 °C, and 30 s annealing and extension at 55 °C. The relative expression levels were calculated by the delta-delta Ct method [44]. Rice actin gene (*LOC_Os03g50885*) was used as internal control. Three biological replicates were employed, and three technical replicates were designed for each sample. Primers used for qPCR are listed in Table S1.

## Data Availability

Data sharing is not applicable.

## Supplementary Materials

The following supporting information can be downloaded at https://www.mdpi.com/article/10.3390/ijms251910778/s1.

## References

[1] Chen R., Deng Y., Ding Y., Guo J., Qiu J., Wang B., Wang C., Xie Y., Zhang Z., Chen J. (2022). Rice functional genomics: Decades’ efforts and roads ahead. Sci. China Life Sci..

[2] Wang B., Smith S.M., Li J. (2018). Genetic regulation of shoot architecture. Annu. Plant Biol..

[3] Komiya R., Ikegami A., Tamaki S., Yokoi S., Shimamoto K. (2008). *Hd3a* and *RFT1* are essential for flowering in rice. Development.

[4] Taoka K., Ohki I., Tsuji H., Furuita K., Hayashi K., Yanase T., Yamaguchi M., Nakashima C., Purwestri Y.A., Tamaki S. (2011). 14-3-3 proteins act as intracellular receptors for rice Hd3a florigen. Nature.

[5] Tsuji H., Tachibana C., Tamaki S., Taoka K., Kyozuka J., Shimamoto K. (2015). Hd3a promotes lateral branching in rice. Plant J..

[6] Wang Y., Li J. (2011). Branching in rice. Curr. Opin. Plant Biol..

[7] Shao G., Lu Z., Xiong J., Wang B., Jing Y., Meng X., Liu G., Ma H., Liang Y., Chen F. (2019). Tiller bud formation regulators MOC1 and MOC3 cooperatively promote tiller bud outgrowth by activating FON1 expression in rice. Mol. Plant.

[8] Takai T. (2024). Potential of rice tillering for sustainable food production. J. Exp. Bot..

[9] Khuvung K., Silva Gutierrez F.A.O., Reinhardt D. (2022). How Strigolactone shapes shoot architecture. Front. Plant Sci..

[10] Dun E.A., Brewer P.B., Gillam E.M.J., Beveridge C.A. (2023). Strigolactones and shoot branching: What is the real hormone and how does it work?. Plant Cell Physiol..

[11] Visentin I., Ferigolo L.F., Russo G., Korwin Krukowski P., Capezzali C., Tarkowská D., Gresta F., Deva E., Nogueira F.T.S., Schubert A. (2024). Strigolactones promote flowering by inducing the miR319-LA-SFT module in tomato. Proc. Natl. Acad. Sci. USA.

[12] Waters M.T., Gutjahr C., Bennett T., Nelson D.C. (2017). Strigolactone signaling and evolution. Annu. Rev. Plant Biol..

[13] Seto Y., Yasui R., Kameoka H., Tamiru M., Cao M., Terauchi R., Sakurada A., Hirano R., Kisugi T., Hanada A. (2019). Strigolactone perception and deactivation by a hydrolase receptor DWARF14. Nat. Commun..

[14] Mostofa M.G., Ha C.V., Rahman M.M., Nguyen K.H., Keya S.S., Watanabe Y., Itouga M., Hashem A., Abd Allah E.F., Fujita M. (2021). Strigolactones modulate cellular antioxidant defense mechanisms to mitigate arsenate toxicity in rice shoots. Antioxidants.

[15] Zhao L.H., Zhou X.E., Yi W., Wu Z., Liu Y., Kang Y., Hou L., de Waal P.W., Li S., Jiang Y. (2015). Destabilization of strigolactone receptor DWARF14 by binding of ligand and E3-ligase signaling effector DWARF3. Cell Res..

[16] Jiang L., Liu X., Xiong G., Liu H., Chen F., Wang L., Meng X., Liu G., Yu H., Yuan Y. (2013). DWARF 53 acts as a repressor of strigolactone signalling in rice. Nature.

[17] Temmerman A., Guillory A., Bonhomme S., Goormachtig S., Struk S. (2022). Masks start to drop: Suppressor of MAX2 1-like proteins reveal their many faces. Front. Plant Sci..

[18] Lin Q., Zhou Z., Luo W., Fang M., Li M., Li H. (2017). Screening of proximal and interacting proteins in rice protoplasts by proximity-dependent biotinylation. Front. Plant Sci..

[19] Liang W.H., Shang F., Lin Q.T., Lou C., Zhang J. (2014). Tillering and panicle branching genes in rice. Gene.

[20] Shalit-Kaneh A., Eviatar-Ribak T., Horev G., Suss N., Aloni R., Eshed Y., Lifschitz E. (2024). The flowering hormone florigen accelerates secondary cell wall biogenesis to harmonize vascular maturation with reproductive development. Proc. Natl. Acad. Sci. USA.

[21] Navarro C., Abelenda J.A., Cruz-Oró E., Cuéllar C.A., Tamaki S., Silva J., Shimamoto K., Prat S. (2011). Control of flowering and storage organ formation in potato by *FLOWERING LOCUS T*. Nature.

[22] Lee R., Baldwin S., Kenel F., McCallum J., Macknight R. (2013). *FLOWERING LOCUS T* genes control onion bulb formation and flowering. Nat. Commun..

[23] Kinoshita T., Ono N., Hayashi Y., Morimoto S., Nakamura S., Soda M., Kato Y., Ohnishi M., Nakano T., Inoue S. (2011). *FLOWERING LOCUS T* regulates stomatal opening. Curr. Biol..

[24] Takeshima R., Nan H., Harigai K., Dong L., Zhu J., Lu S., Xu M., Yamagishi N., Yoshikawa N., Liu B. (2019). Functional divergence between soybean FLOWERING LOCUS T orthologues, FT2a and FT5a, in post-flowering stem growth. J. Exp. Bot..

[25] Li M., Li H. (2023). Breeding of Lotus japonicus that can overcome adverse seasonal environment by coupling flowering time and abiotic stresses. Plant Breed..

[26] Sussmilch F.C., Berbel A., Hecht V., Vander Schoor J.K., Ferrándiz C., Madueño F., Weller J.L. (2015). Pea *VEGETATIVE2* is an *FD* homolog that is essential for flowering and compound inflorescence development. Plant Cell.

[27] Cheng X., Li G., Krom N., Tang Y., Wen J. (2021). Genetic regulation of flowering time and inflorescence architecture by *MtFDa* and *MtFTa1* in *Medicago truncatula*. Plant Physiol..

[28] Zhang P., Liu H., Mysore K.S., Wen J., Meng Y., Lin H., Niu L. (2021). *MtFDa* is essential for flowering control and inflorescence development in *Medicago truncatula*. J. Plant Physiol..

[29] Osnato M., Matias-Hernandez L., Aguilar-Jaramillo A.E., Kater M.M., Pelaz S. (2020). Genes of the RAV family control heading date and carpel development in rice. Plant Physiol..

[30] Liu H., Huang X., Ma B., Zhang T., Sang N., Zhuo L., Zhu J. (2021). Components and functional diversification of florigen activation complexes in cotton. Plant Cell Physiol..

[31] Tylewicz S., Tsuji H., Miskolczi P., Petterle A., Azeez A., Jonsson K., Shimamoto K., Bhalerao R.P. (2015). Dual role of tree florigen activation complex component FD in photoperiodic growth control and adaptive response pathways. Proc. Natl. Acad. Sci. USA.

[32] Sheng X., Hsu C.Y., Ma C., Brunner A.M. (2022). Functional diversification of *Populus FLOWERING LOCUS D-LIKE3* transcription factor and two paralogs in shoot pntogeny, flowering, and vegetative phenology. Front. Plant Sci..

[33] Tsoy O., Mushegian A. (2022). Florigen and its homologs of FT/CETS/PEBP/RKIP/YbhB family may be the enzymes of small molecule metabolism: Review of the evidence. BMC Plant Biol..

[34] Fang Z., Ji Y., Hu J., Guo R., Sun S., Wang X. (2020). Strigolactones and brassinosteroids antagonistically regulate the stability of the D53-OsBZR1 complex to determine FC1 expression in rice tillering. Mol. Plant..

[35] Ashikari M., Sakakibara H., Lin S., Yamamoto T., Takashi T., Nishimura A., Angeles E.R., Qian Q., Kitano H., Matsuoka M. (2005). Cytokinin oxidase regulates rice grain production. Science.

[36] Wang H., Tong X., Tang L., Wang Y., Zhao J., Li Z., Liu X., Shu Y., Yin M., Adegoke T.V. (2022). RLB (RICE LATERAL BRANCH) recruits PRC2-mediated H3K27 tri-methylation on OsCKX4 to regulate lateral branching. Plant Physiol..

[37] Yuan Y., Du Y., Delaplace P. (2024). Unraveling the molecular mechanisms governing axillary meristem initiation in plants. Planta.

[38] Li M., Li H., Li X., Hu X., Pan X., Wu G. (2011). Genetic transformation and overexpression of a rice *Hd3a* induces early flowering in *Saussurea involucrata* Kar. et Kir. ex Maxim. Plant Cell Tiss. Organ Cult..

[39] Ma X., Zhang Q., Zhu Q., Liu W., Chen Y., Qiu R., Wang B., Yang Z., Li H., Lin Y. (2015). A robust CRISPR/Cas9 system for convenient, high-efficiency multiplex genome editing in monocot and dicot plants. Mol. Plant.

[40] Li M., Li H. (2003). A simple and highly efficient *Agrobacterium* mediated rice transformation system. Acta Biol. Exp. Sin..

[41] Komatsu S. (2015). Western blotting using PVDF membranes and its downstream applications. Methods Mol. Biol..

[42] Liu Q., Pante N., Misteli T., Elsagga M., Crisp M., Hodzic D., Burke B., Roux K.J. (2007). Functional association of Sun1 with nuclear pore complexes. J. Cell Biol..

[43] Roux K.J., Kim D.I., Raida M., Burke B. (2012). A promiscuous biotin ligase fusion protein identifies proximal and interacting proteins in mammalian cells. J. Cell Biol..

[44] Pfaffl M.W. (2001). A new mathematical model for relative quantification in real-time RT-PCR. Nucleic Acids Res..

