# Supplementary material for: Heading Date 3a Stimulates Tiller Bud Outgrowth in Oryza sativa L. through Strigolactone Signaling Pathway
Source: Int J Mol Sci. 2024 Oct 7;25(19):10778. doi: 10.3390/ijms251910778 (PMC11476357; doi:10.3390/ijms251910778)
Supplement: Supplementary file 1 [file ijms-25-10778-s001.zip › Table S1.pdf]

Table S1 Primers for gene cloning and qRT-PCR

Hd3a-Fw AATTCATATGATGGCCGGAAGTGGCAGGGACAGG
Hd3a-Re AATTGAATTCTCAGGGGTAGACCCTCCTGCCGCCGGA
D14-Fw AATAGATCTATGCTGCGATCGACGCATCC
D14-Re AATGAATTCCCAAGATTGCCCACATCGTCG
D53-Fw AATGGTCTCGGATCCCTTCACCGGTCAGCGATGCCCACT
D53-Re AATGGTCTCGAATTCATCTAGAATTATTCTTGGCGGGAGCA
qRT-Actin-F CGGTGTCATGGTCGGAAT
qRT-Actin-R GCTCGTTGTAGAAGGTGT
qRT- D3-F CCACCATTTGCTGATTCGTTCT
qRT- D3-R ATGTTCTGATGCTGATCTTGTTCC
qRT- D14-F TCTCCCCGGTTCTTGAACGA
qRT- D14-R CGTCGAACACCTGCTGTATCT
qRT- D53-F TGGGTTCTGAGTGCATGTTG
qRT- D53-R GTGTCCTCACAGGCCACTAG
qRT- D10-F CTGTACAAGTTCGAGTGGCACC
qRT- D10-R CCTCGTCCGTCTCCTCGTAC
qRT- D17-F GAGGATGGTGGCTATGTTCT
qRT- D17-R AGACTGGATCTGATGCTTGCT
qRT- D27-F TCTGGGCTAAAGAATGAAAAGGA
qRT- D27-R AGAGCTTGGGTCACAATCTCG
qRT- FC1-F ATGCTTCCTTTCTTCGATTCCCC
qRT- FC1-R AAGAAGGTGGAATGGTGGTGGTG
