# Supplementary figures and images for: Heading Date 3a Stimulates Tiller Bud Outgrowth in Oryza sativa L. through Strigolactone Signaling Pathway
Source: Int J Mol Sci. 2024 Oct 7;25(19):10778. doi: 10.3390/ijms251910778 (PMC11476357; doi:10.3390/ijms251910778)

## Slide 1
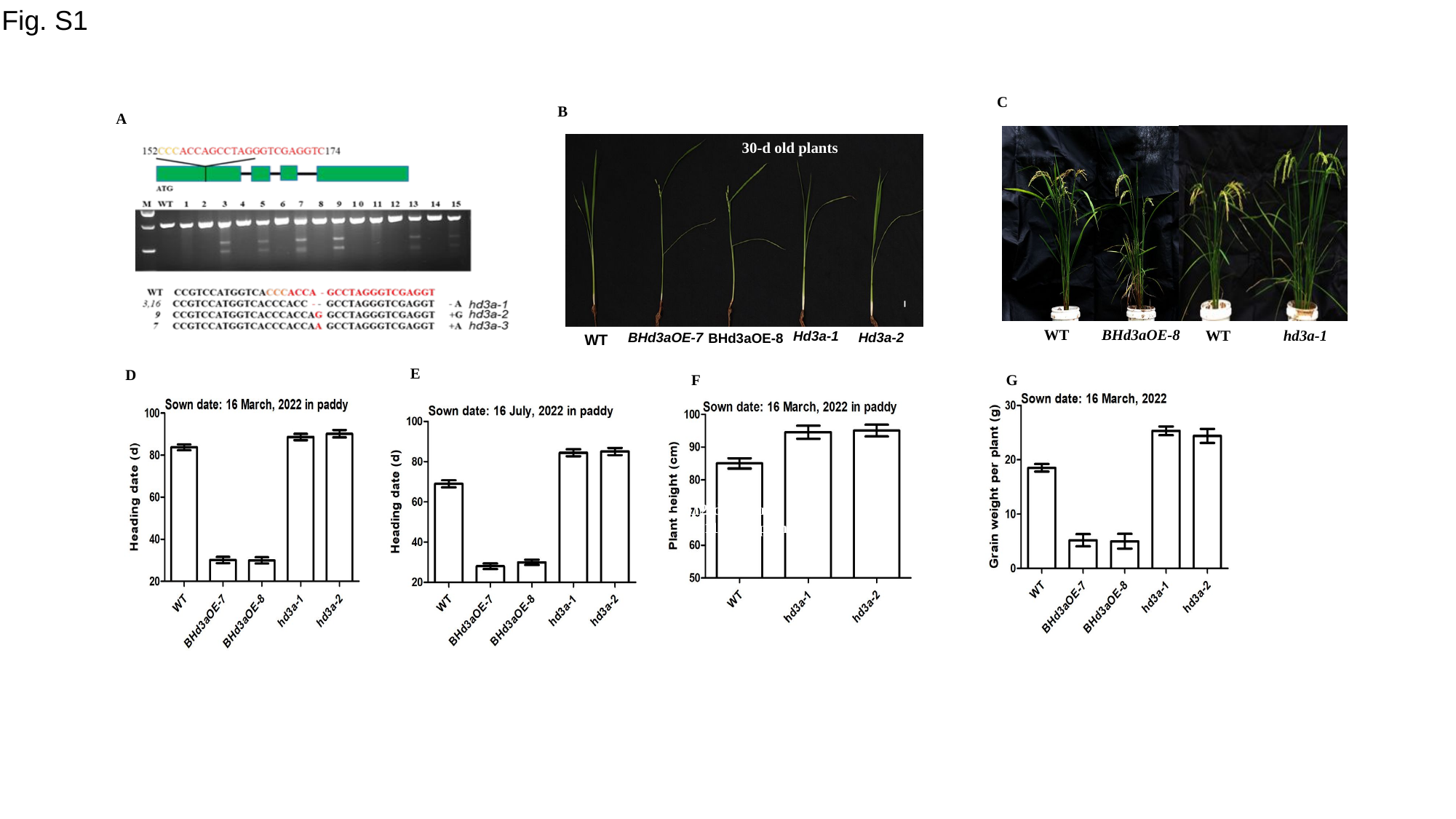

Fig. S1
C
B
A
30-d old plants
BHd3aOE-8
WT
WT
hd3a-1
Hd3a-1
Hd3a-2
BHd3aOE-7
BHd3aOE-8
WT
E
D
F
G
112-day plant
112-day plant

Supplement: Supplementary file 1 [file ijms-25-10778-s001.zip › Fig. S1.pptx]
